# WNT5a-ROR Signaling Is Essential for Alveologenesis

**DOI:** 10.3390/cells9020384

**Published:** 2020-02-07

**Authors:** Changgong Li, Susan M Smith, Neil Peinado, Feng Gao, Wei Li, Matt K Lee, Beiyun Zhou, Saverio Bellusci, Gloria S Pryhuber, Hsin-Yi Henry Ho, Zea Borok, Parviz Minoo

**Affiliations:** 1 Division of Neonatology, Departments of Pediatrics, LAC+USC Medical Center and Childrens Hospital, Los Angeles, CA 90033, USA; susansmi@usc.edu (S.M.S.); nap_698@usc.edu (N.P.); gao241@usc.edu (F.G.); weili1986@yahoo.com (W.L.); mattlee@usc.edu (M.K.L.);; 2 Hastings Center for Pulmonary Research and Division of Pulmonary, Critical Care and Sleep Medicine, Department of Medicine, Keck School of Medicine of USC, Los Angeles, CA 90033, USA; bzhou@usc.edu (B.Z.); zborok@usc.edu (Z.B.); 3 Universities of Giessen and Marburg Lung Center (UGMLC), Justus-Liebig-University Giessen, German Center for Lung Research (DZL), Giessen 35390, Germany; 4 Division of Neonatology, Department of Pediatrics, University of Rochester Medical Center, Rochester, NY 14642, USA; gloria_pryhuber@urmc.rochester.edu; 5 Department of Cell Biology and Human Anatomy, School of Medicine, University of California, Davis, CA 95616, USA; hyhho@ucdavis.edu

**Keywords:** WNT5a, ROR, lung, alveologenesis, secondary crest myofibroblast, migration

## Abstract

WNT5a is a mainly “non-canonical” WNT ligand whose dysregulation is observed in lung diseases such as idiopathic pulmonary fibrosis (IPF), chronic obstructive pulmonary disease (COPD) and asthma. Germline deletion of *Wnt5a* disrupts embryonic lung development. However, the temporal-specific function of WNT5a remains unknown. In this study, we generated a conditional loss-of-function mouse model (*Wnt5a^CAG^*) and examined the specific role of *Wnt5a* during the saccular and alveolar phases of lung development. The lack of *Wnt5a* in the saccular phase blocked distal airway expansion and attenuated differentiation of endothelial and alveolar epithelial type I (AT1) cells and myofibroblasts. Postnatal *Wnt5a* inactivation disrupted alveologenesis, producing a phenotype resembling human bronchopulmonary dysplasia (BPD). Mutant lungs showed hypoalveolization, but endothelial and epithelial differentiation was unaffected. The major impact of *Wnt5a* inactivation on alveologenesis was on myofibroblast differentiation and migration, with reduced expression of key regulatory genes. These findings were validated in vitro using isolated lung fibroblasts. Conditional inactivation of the WNT5a receptors *Ror1* and *Ror2* in alveolar myofibroblasts recapitulated the *Wnt5a^CAG^* phenotype, demonstrating that myofibroblast defects are the major cause of arrested alveologenesis in *Wnt5a^CAG^* lungs. Finally, we show that *WNT5a* is reduced in human BPD lung samples, indicating the clinical relevance and potential role for WNT5a in pathogenesis of BPD.

## 1. Introduction

Structurally and functionally, the mammalian lung represents a remarkably complex and cellularly diverse organ. The primary architecture of the mouse lung as a highly branched structure is laid down during early embryonic days (E) 9.5 through E16.5 by branching morphogenesis. Subsequently, from E17.5 to postnatal day (PN) 4, structures known as saccules are formed that are predecessors of the final respiratory units or alveoli. Alveolar formation, or alveologenesis in the mouse lung occurs entirely in the postnatal period and is estimated to be complete by PN30 [1]. Biological events spanning the late saccular stage to postnatal alveologenesis are required for full maturation of the lung as an efficient gas exchange organ. While a wealth of information is currently available on mechanisms of early lung morphogenesis, our understanding of late embryonic development and postnatal alveologenesis remain surprisingly limited.

During the saccular phase of mouse lung development, distal airways in the lung undergo “expansion” to form saccules. Subsequently, the progenitors of AT1 (NKX2.1^pos^HOPX^pos^) and AT2 (NKX2.1^pos^SFTPC^pos^) cells begin to give rise to mature and functional AT1 (AQP5^pos^PDPN^pos^) and AT2 (SFTPB^pos^SFTPC^pos^) cells [2]. Concurrently, the saccular walls undergo thinning as the capillary networks are established and surfactant production commences. A major event is the formation of structures known as secondary crests (SC), which on 2-dimensional images appear as protrusions into the saccular space [3]. Formation of SC is driven by mesodermally derived secondary crest myofibroblasts (SCMF), also known as alveolar myofibroblasts. Progenitors of SCMF express *Pdgfra* in the saccular phase and *Atca2* in the alveolar phase of lung development [4,5,6]. As they differentiate, SCMF undergo directed migration to form the SC. Disruption of SCMF differentiation or migration leads to arrested alveologenesis [4,5]. Differentiation of the latter cell types during late stage of lung development or lung maturation is associated with expression of several cell-type-specific genes, as listed in Table S1.

WNT signaling is a critical regulator of normal lung morphogenesis, homeostasis and injury repair. WNT ligands can activate the beta-catenin-dependent (the canonical) or the alternative beta-catenin-independent (the non-canonical) pathways [7]. WNT5a, a predominantly non-canonical WNT ligand, is increasingly recognized as an important regulator of stem-cell renewal, cell migration, cell polarity and inflammatory responses [8,9]. WNT5a expression is present in both epithelial and mesenchymal compartments during embryonic stages and is mainly in fibroblasts and endothelial cells in adult lungs [9,10,11]. Dysregulated WNT5a signaling is observed in many lung diseases, ranging from chronic obstructive pulmonary disease (COPD) [12] and idiopathic pulmonary fibrosis (UIP/IPF) [13] to asthma [14]. There are reliable data indicating that exquisitely regulated, temporal and cell-type-specific WNT5a signaling is a strict requirement for normal lung development. We and others have shown that lack of WNT5a activity is associated with abnormal branching of distal airways together with defects in capillaries and alveolar airspaces [10,15]. Conversely, overexpression of *Wnt5a* in *Sftpc-Wnt5a* transgenic mice disrupts epithelial branching and lobe formation but the mice are postnatally viable, indicating no impact on lung function [16]. Importantly however, to date the role of *Wnt5a* in alveologenesis remains unclear.

ROR1 and ROR2 are tyrosine kinase receptors with functional redundancy [17]. Germline deletion of either *Ror1* or *Ror2* disrupts lung development [17]. Compound *Ror1*/*Ror2* mutant mice present with a more severe, abnormal phenotype compared to single gene mutants, and are similar in phenotype to *Wnt5a* knockout mice [17,18]. Both ROR1 and ROR2 mediate WNT5a signaling [18,19,20,21]. In compound transgenic lungs, inactivation of *Ror1* and *Ror2* blocks the lung phenotype caused by overexpression of *Wnt5a* in *Sftpc-Wnt5a* mice [21].

In this study, we generated a number of conditional *Wnt5a* loss-of-function genetic models to elucidate the precise function of *Wnt5a* during specific late stages of lung development. We found that differentiation of multiple cell types was disrupted by conditional *Wnt5a* inactivation in the saccular phase. In contrast, conditional *Wnt5a* inactivation in the alveolar phase had no impact on epithelial cell differentiation, but interrupted differentiation and migration of the mesodermally derived SCMF, leading to defective alveolar formation. Finally, conditional inactivation of the WNT5a receptors *Ror1* and *Ror2* in SCMF also resulted in a similar arrested alveologenesis phenotype, validating our findings on the role of WNT5a signaling during the critical phase of alveolar formation. 

## 2. Materials and Methods

### 2.1. Mouse Breeding and Genotyping

All animals were maintained and housed in pathogen-free conditions at constant room temperature (20–22 °C), with a 12 h light/dark cycle, and free access to water and food in the animal facility of University of Southern California, according to a protocol approved by the USC Institutional Animal Care and Use Committee (IACUC) (Los Angeles, CA, USA). CAG-cre^ER^;Rosa26^mTmG^;Wnt5a^f/f^ (Wnt5a^CAG^) mice were generated by breeding CAG-cre^ER^ mice (Tg(CAG-cre/Esr1*)5Amc/J, The Jackson Laboratory) and Rosa26^mTmG^ (Gt(ROSA)26Sor^tm4(ACTB-tdTomato,-EGFP)Luo^/J, The Jackson Laboratory) mice with Wnt5a^f/f^ mice [22]. Gli1-cre^ERT2^;Rosa26^mTmG^ mice (mTmG^Gli^) were generated by breeding Gli1-cre^ERT2^ [23] and Rosa26^mTmG^ mice. Gli1-cre^ERT2^;Rosa26^mTmG^;Ror1^f/f^;Ror2^f/f^ (Ror^Gli;GFP^) mice were generated by breeding mTmG^Gli^ mice with the Ror1^f/f^;Ror2^f/f^ mice [18].

Genotyping of the transgenic mice was performed by PCR with genomic DNA isolated from mouse tails. The forward (F) and reverse primers (R) for transgenic mouse genotyping are listed below.

*CAG-cre^ER^:* (forward) 5’-TAAAGATATCTCACGTACTGACGGTG-3’ and (reverse) 5’-TCTCTGACCAGAGTCATCCTTAGC-3’.

*Gli1-cre^ERT2^*: (forward) 5’-TAA AGA TAT CTC ACG TAC TGA CGG TG-3’ and (reverse) 5’-TCT CTG ACC AGA GTC ATC CTT AGC-3’. 

*Wnt5a^f/f^*: (Forward) 5’-TGAGGGACTGGAAGTTGCAGG-3’, and (Reverse) 5’-TTCCAATGGGCTTCTGGAGAG-3’.

*Ror1^f/f^*: (Forward) 5’-CGTTTTCCTCCTGCTCACAGG-3’, and (Reverse) 5’-GCAGACCTGGTGAATTCTACCTCAG-3’.

*Ror2^f/f^*: (Forward) 5’-CATGGCCACATCCATACCGAG-3’, and (Reverse) 5’-CCGGCGAGCGTGCTTAGATAGCCC-3’.

### 2.2. Tamoxifen Administration

For embryonic studies, tamoxifen (Sigma, 40mg/mL in peanut oil) was administered by oral gavage to pregnant females from E14 to E17 (2.4 mg each mouse, one dose per day). Embryonic lungs were collected at E18. For neonatal studies, tamoxifen (8mg/mL in peanut oil) was administered by oral gavage to neonates at postnatal day 2 (P2, 400 μg each pup) with a plastic feeding needle (Instech Laboratories, PA). Neonatal lungs were collected between P12 and P13 for morphological, immunohistochemical and molecular biological analyses.

### 2.3. Human Neonatal Lung Samples

Human lung tissue samples were obtained postmortem by expedited autopsy of preterm infants having the diagnoses of mild RDS, evolving and established BPD as well as term infants as controls (no lung disease). Consented collection and processing of the lung samples as from the deceased were approved by the University of Rochester Institutional Review Board. Selected clinical and recovery details have been previously published [24].

### 2.4. Immunofluorescent Staining

Immunofluorescent staining was conducted as previously described [25] using paraffin-embedded lung sections. In brief, five micrometer (μm) tissue sections were deparaffinized, rehydrated and subjected to antigen retrieval. After blocking with normal serum, the sections were probed with primary antibodies at 4 °C overnight. Combinations of fluorescein anti-mouse and Cy3 anti-rabbit or anti-goat IgG secondary antibodies (Jackson ImmunoResearch Laboratories, ING) were applied to detect specific primary antibodies. Nuclei were counterstained with 4′,6-diamidino-2-phenylindole (DAPI). Primary antibodies used are listed in Table S3.

### 2.5. Neonatal Lung Fibroblast Isolation, Treatment and Migration Assay

P5 neonatal lungs of *Wnt5a^CAG^* mice and littermate controls were dissected in HBSS (GIBCO24020-117), inflated with Dispase and digested by continuous shaking in Dispase at 37 °C for 15 min. Lung lobes were then isolated, cut into small pieces, transferred into Miltenyi tubes in 5 mL HBSS and dissociated with a gentle MACS dissociator (Miltenyi Biotec. Inc., San Diego, CA, USA). After filtering through 40 μm cell strainers, the dissociated cells were collected by centrifugation at 1200 rpm for 5 min and resuspended in DMEM containing 10% FBS. The cells were then plated in cell-culture plates and incubated at 37 °C with 5% CO_2_ for 1 h. After removing floating cells, the attached fibroblasts were washed with PBS and cultured in fresh medium. Loosely attached cells were removed by repeated washes within 20 h of culture. The purity of the fibroblasts isolated under this condition was examined by using the *Pdgfra-EGFP* model, which expresses enhanced GFP under the control of an endogenous *Pdgfra* promoter [26]. Approximately 90% of the attached cells were GFP positive 20 h after cell isolation. When the cells grew to near confluence, they were trypsinized and stored in 10% DMSO (DMEM + 10% FBS + 10% DMSO) in liquid nitrogen for future use. For the cell migration assay, cells grown in culture flasks (ThermoFisher Scientific, Waltham, MA, USA) were treated with tamoxifen (1.5 μg/mL) for 24 h, and then re-plated (1 × 10^5^ cells per Transwell) onto 12 µm Transwell membranes (MiliporeSigma, Burlington, MA, USA) and cultured in DMEM containing 1% FBS inside and 10% FBS outside the Transwell insert. After 24 h, the cells were fixed in 4% formaldehyde, washed with PBS and stained with crystal violet. The cells on the upper side of the membrane were cleaned with a cotton swab and the cells that migrated to the lower side of the Transwell membrane were imaged and counted.

### 2.6. Real-time Quantitative Polymerase Chain Reaction (real-time RT-PCR)

Neonatal mouse lung fibroblasts were isolated and cultured as described above (Section 2.5). 1.5x10^5^ cells were plated onto a 12-well culture plate until they grew to near confluence. The cells were treated with tamoxifen (1.5 μg/mL) for 24 h and then collected for RNA isolation with TRIzol reagent (ThermoFisher Scientific, MA, USA). cDNA was synthesized with an EasyScript Plus cDNA synthesis kit according to the manufacturer’s protocol (Lamda Biotech Inc., Ballwin, MO, USA). Expression of selected genes was quantified by real-time RT-PCR using a LightCycler with LightCycler Fast Start DNA Master SYBR Green I Kit (Roche Applied Sciences, Indianapolis, IN, USA) as previously described [16]. Each reaction contained 3 μL of FastStart SYBR green reaction mix plus enzyme, 2.5 µM of primers and 0.5 µL of cDNA in a total volume of 20 μL. The running protocol consisted of four steps, including 1) denaturation at 95 °C for 6 min; 2) amplification and quantification under 95 °C for 0–10 s, 62 °C for 15 s and 72 °C for 20 s for 45 cycles; 3) melting curve program, where the reaction temperature rapidly increases to 95 °C, then decreases to 60 °C for 15 s, followed by a slow increase to 98 °C at a rate of 0.1 °C per s with continuous fluorescence monitoring; and 4) cooling to 40 °C. Reaction conditions were optimized to create a one-peak melting curve. The ΔΔCt method was used to calculate relative ratios of a target gene mRNA in mutant lungs compared to littermate control lungs. TBP (TATA-Box Binding Protein) was used as the reference gene. Primers for real-time RT-PCR were designed using the program of the Universal ProbeLibrary Assay Design Center from Roche Applied Sciences (IN). Sequences of the primers are listed in Table S4.

### 2.7. Statistics Analysis 

At least three biologically independent control and mutant lungs were used for each morphometric analysis and real-time RT-PCR analyses. Five to six images (10× magnification) from each lung were used to manually count MLI. Quantitative data are presented as mean values ± the standard error of the mean. *p* values were calculated by two-tailed Student’s *t* tests. 

## 3. Results 

### 3.1. WNT5a is Required for Differentiation of Multiple Cell Types During Saccular Stage Lung Development

**Figure 1:**
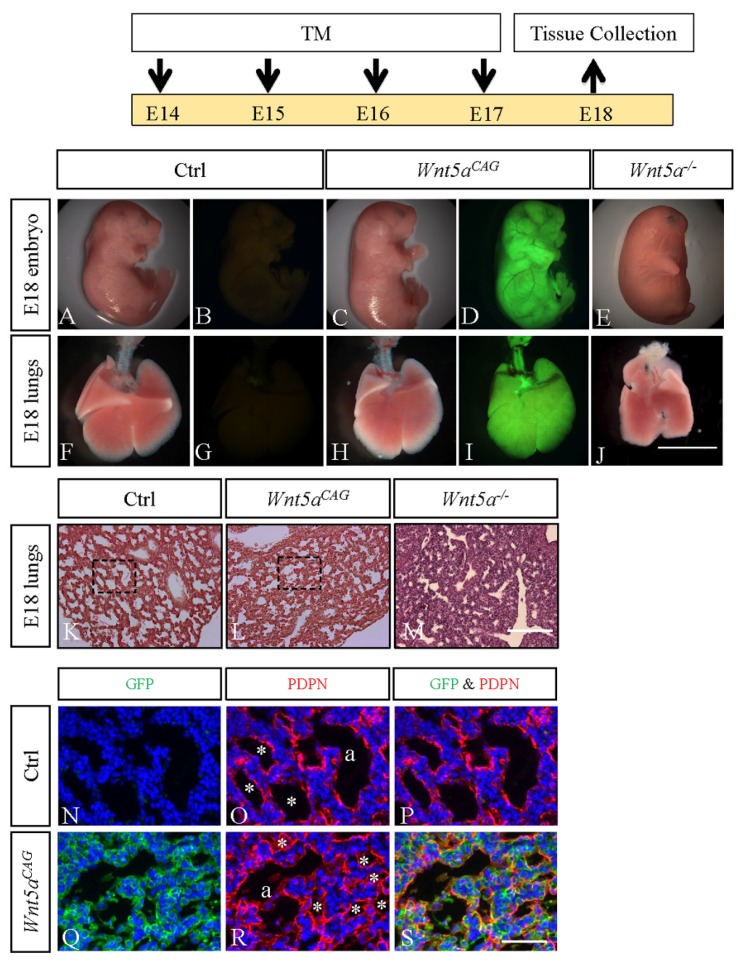
*Wnt5a* inactivation attenuated saccular stage lung development. Top: Schematic of the experimental plan. **A**–**E**: Gross morphology of E18 control (**A** and **B**), *Wnt5a^CAG^* (**C** and **D**) and *Wnt5a^-/-^* (**E**) embryos. Panels **A**, **C** and **E** show the bright-field images. Panels **B** and **D** show the green fluorescent images of control and *Wnt5a^CAG^* embryos, respectively. **F**–**J:** Gross morphology of E18 control (**F** and **G**), *Wnt5a^CAG^* (**H** and **I**) and *Wnt5a^-/-^* (**J**) lungs. Panels **F**, **H** and **J** show the bright-field images. Panels **G** and **I** show the green fluorescent images of control and *Wnt5a^CAG^* lungs, respectively. Scale bars (**J**): 10 mm for **A**–**E**, 4 mm for **F**–**J**. **K**–**M**: H&E staining of E18 control (**K**), *Wnt5a^CAG^* (**L**) and *Wnt5a^-/-^* (**M**) lungs. Scale bar (M): 200 μm for K–M. **N**–**S**: Immunostaining of GFP (green) and PDPN (red) in E18 control (**N**–**P**) and *Wnt5a^CAG^* (**Q**–**S**) lungs. Panels **N**–**P** and **Q**–**S** correlate to the boxed areas in Panels **K** and **L**, respectively. Asterisk indicates saccules. “a” indicates the junction between bronchiole and saccules. Scale bars (**S**): 50 μm for **N**–**S**.

We previously reported that germline deletion of *Wnt5a* (*Wnt5a^-/-^)* disrupts development of saccular stage lungs [10]. However, whether the lung saccular defects (E18) were secondary consequences of defects that may have occurred during branching morphogenesis was not addressed. In the present study, we generated a regulatable (conditional) triple transgenic mouse model, *CAG-cre^ER^*;*Wnt5a^f/f^;Rosa26^mTmG^*, referred to as *Wnt5a^CAG^* (see Materials and Methods) to target *Wnt5a* globally upon activation of *CAG-cre^ER^* by administration of tamoxifen. To assess the impact of *Wnt5a* inactivation during the saccular stage of lung development, pregnant *Wnt5a^f/f^*;*Rosa26^mTmG^* female mice, mated with *Wnt5a^CAG^*males, received tamoxifen orally from E14 to E17 (Figure 1). The embryos and the isolated lungs were analyzed at E18. As shown in Figure 1, *Wnt5a^f/f^;Rosa26^mTmG^* control (Panels A and B) embryos showed a normal morphology, and *Wnt5a^CAG^* littermate embryos (Panels C and D) were similar to control embryos with the exception of minor craniofacial differences (i.e., mouth) (Figure S1). In contrast, the germline *Wnt5a^-/-^* embryos (Figure 1, Panel E) are profoundly abnormal with truncation of multiple structures, including limbs, tail and mouth [27]. Further analysis showed that compared to *Wnt5a^f/f^;Rosa26^mTmG^* controls (Panels F and G), gross morphology of the littermate *Wnt5a^CAG^*lungs was unaffected by a lack of WNT5a signaling during the saccular phase of lung development (Panels H and I). In contrast, germline *Wnt5a^-/-^* lung lobes were clearly deformed (Panel J) [10]. Histological analysis using hematoxylin and eosin (H&E)-stained multiple lung sections showed that *Wnt5a^f/f^;Rosa26^mTmG^* control lungs (Panel K) are composed of uniformly organized and expanded saccular structures, while the *Wnt5a^CAG^*lungs are heterogenous and consist of both areas of expanded (i.e., sacculi) as well as unexpanded airspace (Figure 1, Panel L). The germline *Wnt5a^-/-^*lungs contained nearly uniformly unexpanded airspace (Figure 1, Panel M). The phenotypic differences between the control and *Wnt5a^CAG^*lungs were further illustrated by immunostaining for GFP and AT1 cell marker PDPN (Figure 1, Panels N–S). 

**Figure 2:**
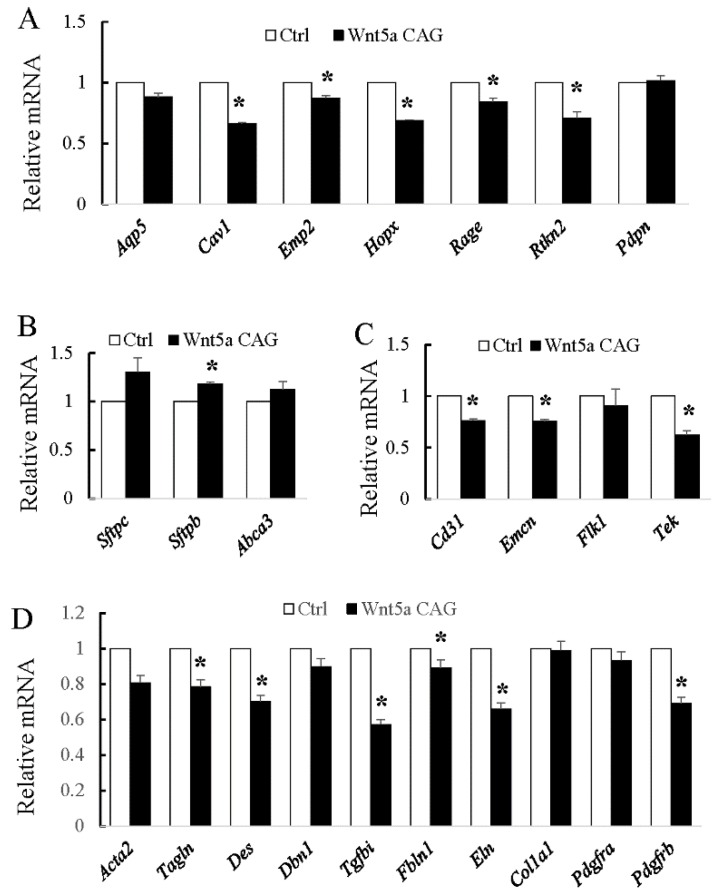
*Wnt5a* inactivation attenuates differentiation of AT1 and endothelial cells and myofibroblasts during the saccular stage of lung development. **A**–**D:** Relative mRNA for AT1 (**A**), AT2 (**B**), endothelial (**C**) and myofibroblast (**D**) markers in E18 *Wnt5a^CAG^* lungs (Wnt5a CAG) compared to control lungs by real-time RT-PCR analysis. Data represent the mean ± standard error of the mean (SEM). * indicates *p* < 0.05. *n* = 3.

Our previous analysis found that AT1 cell differentiation was reduced in *Wnt5a^-/-^* lungs [28]. The saccular phase is the critical time period during which major decisions on establishment of AT1 and AT2 cell lineages as well as differentiation of various mesodermally derived cells take place. We therefore used our conditional *Wnt5a^CAG^* mice in combination with lineage-specific markers for AT1, AT2 and endothelial cells, as well as for myofibroblasts (Table S1) to determine the role of WNT5a specifically during the saccular phase of lung development. As shown in Figure 2, conditional *Wnt5a* inactivation in *Wnt5a^CAG^* embryos during the E14→E17 saccular transition period decreased expression of multiple AT1 *(Cav, Emp2, Hopx, Rage and Rtkn2*), but not AT2 cell markers. This indicates that differentiation of AT1 cells, which are derived from NKX2.1^pos^HOPX^pos^ AT1 progenitors is exquisitely sensitive to loss of WNT5a signaling. In the endothelial lineage, multiple markers were decreased in the mutant lungs (*Cd31, Emcn and Tek*). In addition, many genes related to myofibroblast cell differentiation and function were significantly decreased in *Wnt5a^CAG^* lungs. Therefore, *Wnt5a* signaling is a critical regulator of differentiation for multiple cell types during the saccular phase of lung development.

### 3.2. WNT5a is Required for Myofibroblast Differentiation and Migration During Alveolar Stage Lung Development

**Figure 3:**
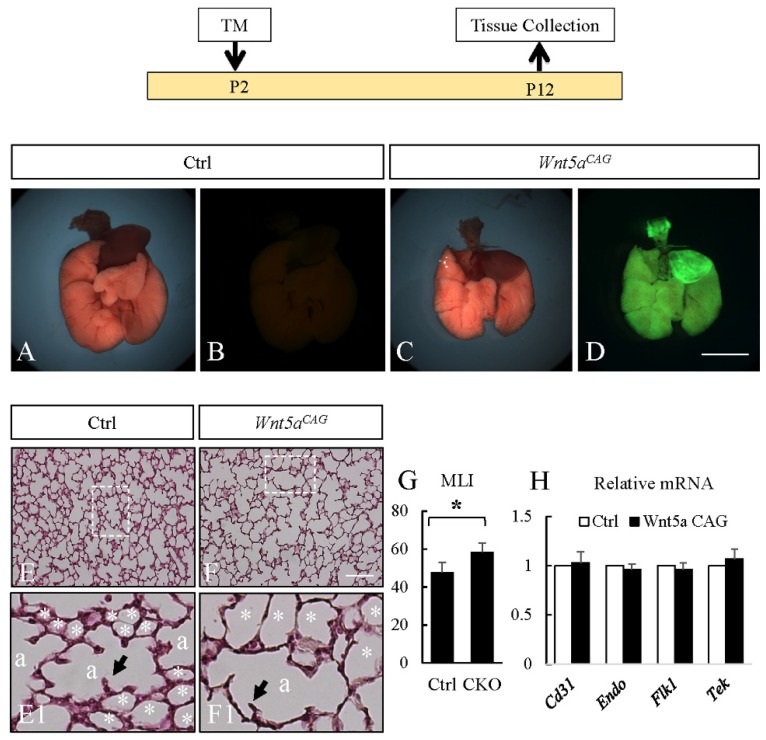
Postnatal inactivation of *Wnt5a* attenuates alveologenesis. Top: Schematic of the experimental plan. **A**–**D:** Gross morphology of P12 control (**A**–**B**) and *Wnt5a^CAG^* (**C**–**D**) lungs. Panels A and C show bright-field images. Panels B and D show green fluorescent images of control and *Wnt5a^CAG^* embryos, respectively. Scale bars (**D**): 4 mm for **A**–**D**. **E**–**F:** H&E staining of P12 control (**E**) and *Wnt5a^CAG^* (**F**) lungs. **E1** and **F1** show higher magnification views of boxed areas (rotated 90 degree clockwise in E1) in E and F, respectively. Please note that distal alveolar units (*) surrounding the dividing alveolar space (a) are profoundly enlarged in the mutant lungs. Arrows indicate 2nd crests. Scale bars (**F**): 100 μm for **E** and **F**; 25 μm for E1 and F1. **G**: Mean linear intercept (MLI) of control and *Wnt5a^CAG^* lungs (CKO). **H**: Relative mRNA for endothelial markers in *Wnt5a^CAG^* lungs (Wnt5a CAG, P12-13) compared to the littermate control lungs by real-time RT-PCR analysis. Data represent the mean ± SEM. * indicates *p* < 0.05. *n* = 4.

**Figure 4:**
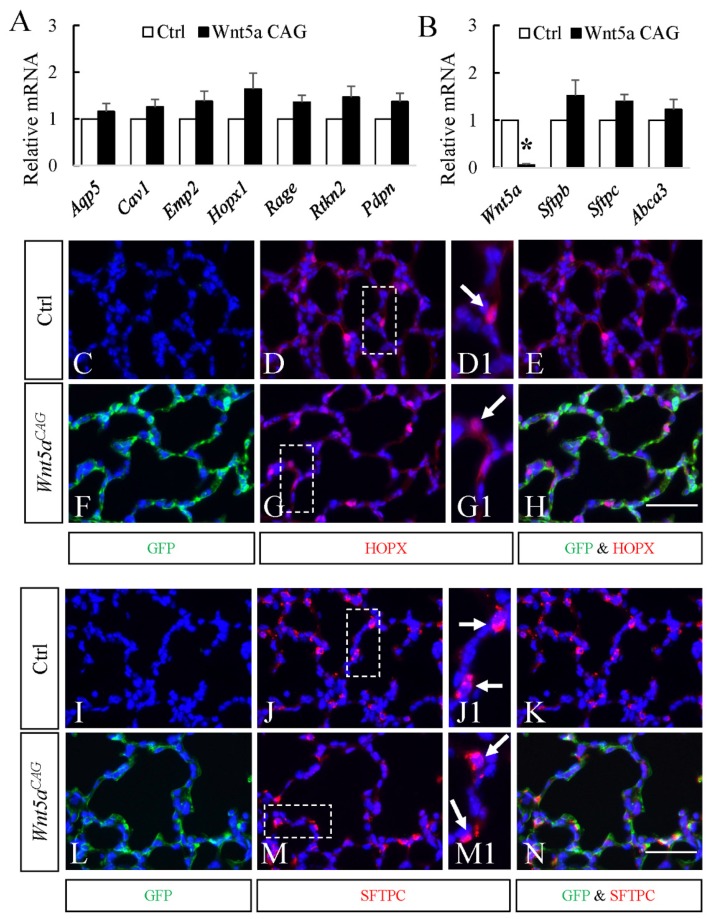
No significant changes in AT1 and AT2 cell markers in P12 *Wnt5a^CAG^* lungs. **A**–**B:** Relative mRNA for AT1(**A**) and AT2 (**B**) cell markers in *Wnt5a^CAG^* lungs (Wnt5a CAG, P12-13) compared to littermate control lungs by real-time RT-PCR analysis. **C**–**H:** Immunostaining of GFP (green) and HOPX (red) in P12 control (**C**, **D**, **D1** and **E**) and *Wnt5a^CAG^* (**F**, **G**, **G1** and **H**) lungs. **D1** and **G1** show higher magnification views of boxed areas in D and G, respectively. Arrows indicate HOPX-positive cells. Scale bar (**H**): 50 μm for **C**, **D**, **E**, **F**, **G** and **H**; 25 μm for **D1** and **G1**. **I**–**N:** Immunostaining of GFP (green) and SFTPC (red) in P12 control (**I**, **J**, **J1** and **K**) and *Wnt5a^CAG^* (**L**, **M**, **M1** and **N**) lungs. **J1** and **M1** show higher magnification views of boxed areas (rotated 90 degree clockwise in **M1**) in **J** and **M**, respectively. Arrows indicate SFTPC-positive cells. Scale bar (**N**): 50 μm for **I**, **J**, **K**, **L**, **M** and **N**; 25 μm for **J1** and **M1**. Data represent the mean ± SEM. * indicates *p* < 0.05. *n* = 4.

Because germline *Wnt5a^-/-^* mice do not survive postnatally, the role of WNT5a in alveolar formation has remained unknown. To address this issue, we conditionally inactivated *Wnt5a* globally at the onset of postnatal alveolar development in *Wnt5a^CAG^* mice by administering tamoxifen on postnatal day 2 (P2). P2 inactivation of *Wnt5a* disrupted alveologenesis resulting in enlarged alveolar structures, reminiscent of a phenotype characteristic of the human neonatal chronic lung disease known as bronchopulmonary dysplasia (BPD). Morphometric quantification showed a significantly increased mean linear intercept, MLI, a measure of alveolar dimensions [29,30]. Analysis of cell differentiation showed no changes in expression of endothelial markers (Figure 3), nor in AT1 and AT2 cell markers (Figure 4). To determine whether the relative proportion of AT1 and AT2 cells was altered, we analyzed lung tissue immunostained for AT1 (HOPX) and AT2 (SFTPC) cell markers (Figure 4). The relative ratio of HOPX^pos^ cells over total DAPI^pos^ cells was not significantly altered in the *Wnt5a^CAG^* lungs (Control: 6.5% ± 1.1%; Mutant: 5.9% ± 1.0%, *p* = 0.69). Similarly, no significant difference was observed in the ratio of SFTPC^pos^ cells (Control: 7.9% ± 0.8%; Mutant: 9.5% ± 1.3%, *p* = 0.30). 

**Figure 5:**
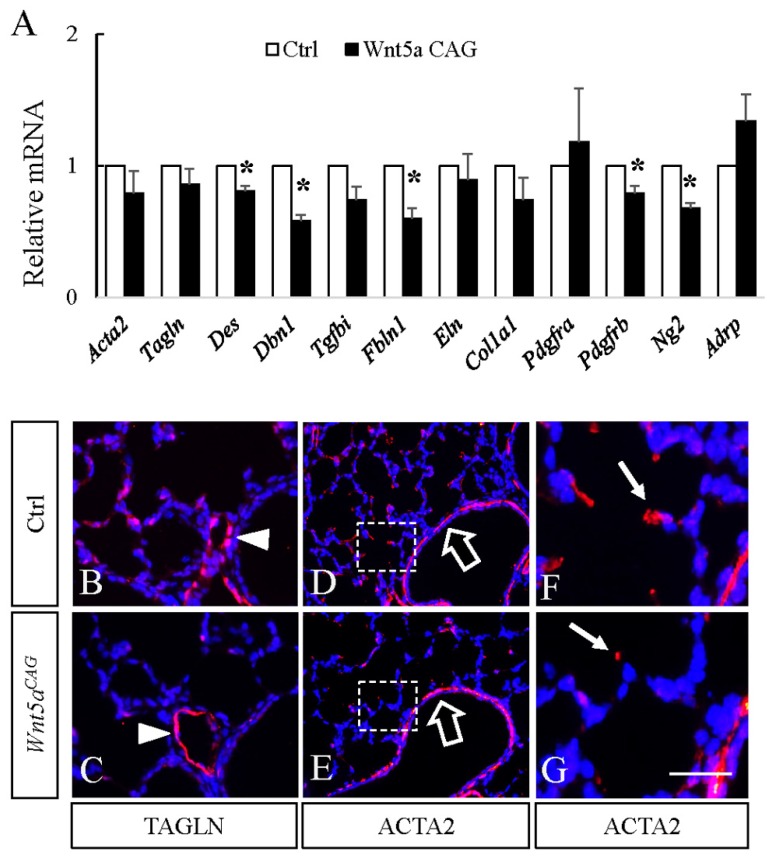
Postnatal inactivation of *Wnt5a* decreases expression of genes enriched in myofibroblasts. **A**: Relative mRNA for genes enriched in myofibroblasts in *Wnt5a^CAG^* lungs (Wnt5a CAG, P12-13) compared to the littermate control lungs by real-time RT-PCR analysis. Data represent the mean ± SEM. * indicates *p* < 0.05. *n* = 4. **B**–**G:** Immunostaining of TAGLN (**B** and **C**) and ACTA2 (**D**–**G**) in P12 control (**B**, **D** and **F**) and *Wnt5a^CAG^* (**C**, **E** and **G**) lungs. Panels **F** and **G** show higher magnification views of boxed areas in **D** and **E**, respectively. Nuclei are counterstained with DAPI. Arrowheads in **B** and **C** indicate TAGLN-positive perivascular smooth muscle cells. Block arrows in **D** and **E** indicate ACTA2 positive parabronchial smooth muscle cells. Arrows in **F** and **G** indicate ACTA2 positive alveolar myofibroblast cells. Scale bars (**G**): 50 μm for **B** and **C**, 100 μm for **D** and **E**, 25 μm for **F** and **G**.

In contrast, multiple markers related to myofibroblast differentiation were reduced (*Des*: 0.81 ± 0.04, *p* < 0.05; *Dbn1*: 0.58 ± 0.05, *p* < 0.05; *Tgfbi*: 0.74 ± 0.10, *p* = 0.07; *Fbln1*: 0.60 ± 0.08, *p* < 0.05). Consistent with the latter data, immunostaining with antibodies against TAGLN and ACTA2, two markers of myofibroblast differentiation, revealed the number of TAGLN^pos^ and ACTA2^pos^ cells in the alveolar region was decreased in the *Wnt5a* mutant lungs (Figure 5). In control lungs, ACTA2^pos^ cells were elongated, having a stretched cell shape. In contrast, ACTA2 staining was condensed in *Wnt5a^CAG^* lungs. Both ACTA2 and TAGLN are expressed in alveolar myofibroblasts. We used TAGLN, which shows stronger perinuclear staining, to evaluate the abundance of alveolar myofibroblasts and found that the ratio of TAGLN^pos^ cells is significantly decreased in *Wnt5a^CAG^* lungs (control: 12.7% ± 0.7%, mutant: 8.0% ± 0.7%, *p* < 0.05). However, TAGLN and ACTA2 staining around the blood vessels and airways appeared similar between the control and mutant lungs. 

**Figure 6:**
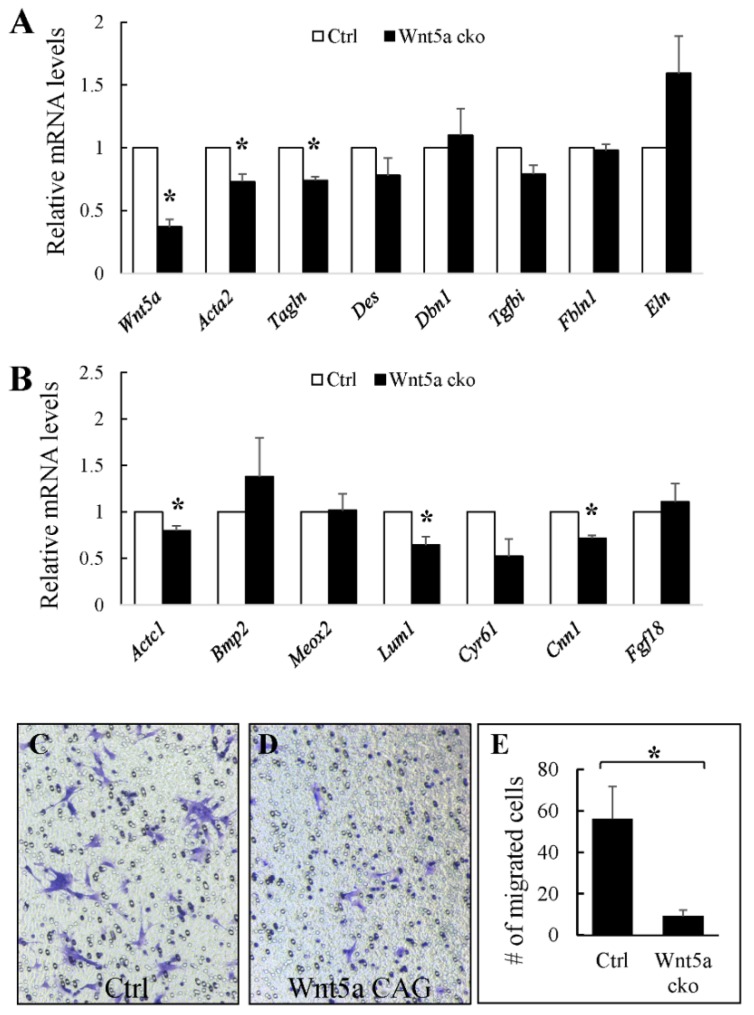
Inactivation of *Wnt5a in vitro* disrupts differentiation and migration of cultured fibroblasts. Fibroblasts from P5 neonatal lungs were isolated, cultured and treated with tamoxifen (1.5 μg/mL) for 24 h before RNA isolation. **A**: Relative mRNA for myofibroblasts-enriched genes in *Wnt5a^CAG^* fibroblasts compared to control lung fibroblasts determined by real-time RT-PCR analysis. **B**: Relative mRNA of cell migration genes in *Wnt5a^CAG^* fibroblasts compared to control lung fibroblasts determined by real-time RT-PCR analysis. Data represent the mean ± SEM. * indicates *p* < 0.05. *n* = 4. **C**–**D**: Transwell migration assay. Control (**C**) and *Wnt5a^CAG^* (**D**) fibroblasts treated with tamoxifen (1.5 μg/mL, 24 h) were plated on 12-micron Transwell membranes and cultured for 24 h. Cells migrated to the lower side of the Transwell were stained with crystal violet and counted. **E**: Quantification of the number of cells that migrated to the lower side in unit area. Four areas from each experiment (each Transwell) were imaged, counted and the means calculated. Date represent the mean of three independent experiments ± SEM. * indicates *p* < 0.05.

The above data indicate that WNT5a signaling during the alveolar phase is required for myofibroblast differentiation. To confirm this finding, we conducted in vitro analyses using fibroblasts isolated from P5 neonatal *Wnt5a^CAG^*and littermate *Wnt5a^f/f^;Rosa26^mTmG^* (control) lungs. We activated CAG-cre^ER^ by treating cultured cells with tamoxifen for 24 hrs. As shown in Figure 6, several myofibroblast genes that were decreased in vivo were also reduced in cultured *Wnt5a* mutant cells, compared to the controls. These included *Acta2* (0.73 ± 0.06, *p* < 0.05), *Tagln* (0.74 ± 0.03, *p* < 0.05) and *Tgfbi* (0.79 ± 0.07, *p* = 0.06). These data support a necessary role for WNT5a signaling in directing differentiation of myofibroblast progenitors in the early postnatal period. 

We recently showed that conditional targeting of *Pdgfra* in SCMF during alveologenesis also disrupts alveolar formation resulting in a phenotype similar to that of *Wnt5a^CAG^* lungs [25]. RNA-seq analysis of the *Pdgfra* mutant SCMF revealed cell migration as one of the top affected cellular functions, reflected as altered expression of multiple cell migration genes [25]. In the present study, we selected several cell migration genes that were disrupted in the *Pdgfra* mutant SCMF (Table S2) and analyzed their expression in fibroblasts isolated from P5 control and *Wnt5a^CAG^* lungs. As shown in Figure 6, we found decreased expression of *Actc1* (0.80 ± 0.05, *p* < 0.05), *Lum1* (0.64 ± 0.09, *p* < 0.05), *Cyr61* (0.53 ± 0.18, *p* = 0.08) and *Cnn1* (0.72 ± 0.03, *p* < 0.05) in *Wnt5a* mutant cells, suggesting WNT5a plays a role in lung myofibroblast migration. To further test this possibility, we examined the migration of control and *Wnt5a^CAG^*fibroblasts in vitro using the Transwell cell culture. As shown in Figure 6, many control fibroblasts migrated to the lower side of the Transwell after 24 h of culture. In contrast, we noted only a few trans-migrated mutant fibroblasts indicating defective migration of *Wnt5a^CAG^* myofibroblasts (control: 56.2 ± 15.5, mutant: 9.2 ± 2.9 in unit area).

### 3.3. RORs are Essential for Myofibroblast Migration During Alveolar Stage Lung Development

**Figure 7:**
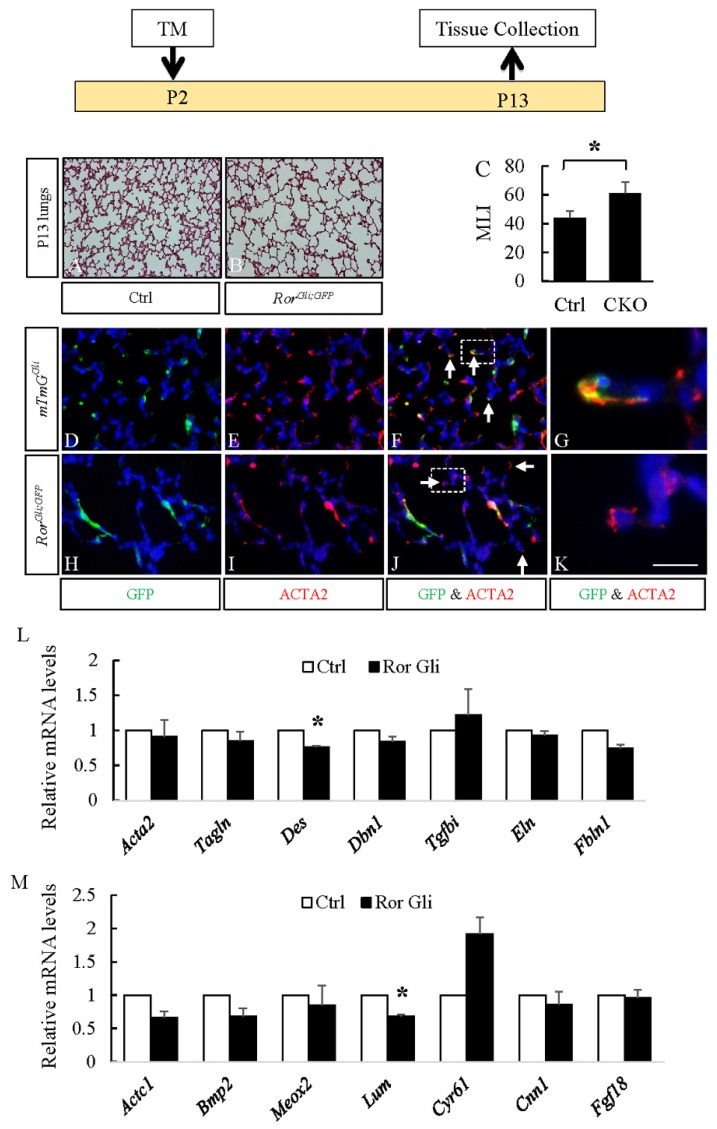
Postnatal inactivation of *Ror1* and *Ror2* attenuated alveologenesis. Top: Schematic of the experimental plan. **A**–**B**: H&E staining of P13 control (**A**) and *Ror^Gli;GFP^* (**B**) lungs. **C**: Mean linear intercept (MLI) of P13 control and *Ror^Gli;GFP^* (CKO) lungs. **D**–**K**: Immunostaining of GFP (green) and ACTA2 (red) in P13 *mTmG^Gli^* (**D**–**G**, control) and *Ror^Gli;GFP^* (**H**–**K**) lungs. Panels **G** and **K** show higher magnification views of boxed areas in F and J, respectively. Nuclei are counterstained with DAPI. Scale bars (**K**): 200 μm for **A** and **B**, 50 μm for **D**–**F** and **H**–**J**, 11 μm for **G** and **K. L**–**M**: Relative mRNA for myofibroblast enriched genes and cell migration genes in P13 *Ror^Gli;GFP^* (Ror Gli) lungs compared to control lungs by real-time RT-PCR analysis. Data represent the mean ± SEM. * indicates *p* < 0.05. *n* = 3.

The sum of the above observations indicates that myofibroblasts are a major target of WNT5a signaling and suggest that defects in this cell lineage may be the major cause of abnormal alveologenesis in *Wnt5a^CAG^* lungs. To test this potential mechanism, we blocked WNT5a signaling by targeting its receptors *Ror1* and *Ror2* in SCMF using *Gli1-cre^ERT2^*. Previously we showed that *Gli1-cre^ERT2^* targets SCMF during mouse alveologenesis [6,25,31]. We therefore generated mice with the compound genotype, *Gli1-cre^ERT2^;Rosa26^mTmG^;Ror1^f/f^;Ror2^f/f^ (Ror^Gli;GFP^)* and activated the *Gli1-cre^ERT2^* by tamoxifen on P2. Lungs from *Ror^Gli;GFP^* and *Rosa26^mTmG^;Ror1^f/f^;Ror2^f/f^* control neonatal mice were characterized on P13 (Figure 7). Postnatal inactivation of *Ror1* and *Ror2* in SCMF resulted in a phenotype nearly identical to that of postnatal *Wnt5a* inactivation in *Wnt5a^CAG^* mice. Characterization of *Ror^Gli;GFP^* lungs showed profound enlargement of alveoli, indicating disruption of alveolar formation. To determine the impact of *Ror* inactivation in the *Gli1-cre^ERT2^*targeted cells, we conducted double immunostaining for GFP and ACTA2 in *Ror^Gli;GFP^* and *Gli1-cre^ERT2^;Rosa26^mTmG^* (*mTmG^Gli^*, control) lungs. Consistent with our previous observations, *Gli1-cre^ERT2^* labeled mainly SCMF that are scattered within the alveolar region and are ACTA2^pos^ at this time point. The majority of GFP^pos^ cells in the control lungs were localized to the tip of the SC. In *Ror^Gli;GFP^* lungs, the majority of GFP^pos^ cells were still localized within the primary septa, indicating that lack of *Ror1* and *Ror2* decreased myofibroblast cell migration in the *Ror^Gli;GFP^* lungs. Consistent with this conclusion, expression of the myofibroblast genes *Des* (0.76 ± 0.02, *p* < 0.05) and *Fbln1* (0.74 ± 0.06, *p* = 0.05) and cell migration genes *Actc1* (0.66 ± 0.10, *p* = 0.07), *Bmp2* (0.68 ± 0.12, *p* = 0.11) and *Lum* (0.68 ± 0.03, *p* < 0.05) were collectively decreased in the *Ror^Gli;GFP^* lungs. Thus, signaling thru ROR1/ROR2, the cognate WNT5a receptors is required for normal migration of SCMF and the process of alveologenesis. 

### 3.4. Relevance to Human Neonatal Lung Disease

**Figure 8:**
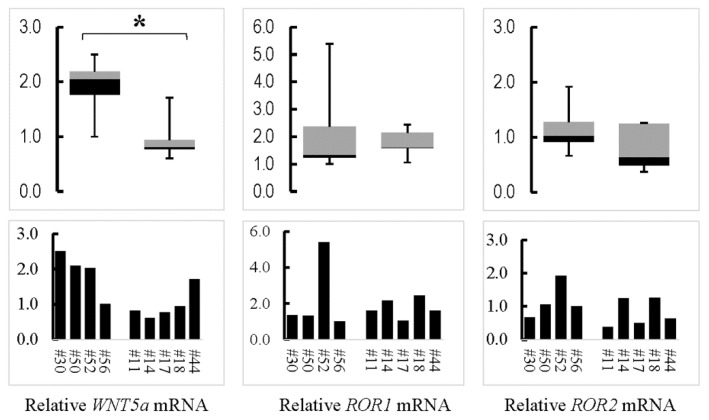
Expression of *WNT5a, ROR1* and *ROR2* in human bronchopulmonary dysplasia (BPD) lungs. Human lung samples from deceased donors were collected and processed at the University of Rochester following the protocol approved by University of Rochester Institutional Review Board. Relative mRNA for the examined genes is presented in a 2-D column chart with levels of #56 arbitrarily set as “1”. Data distribution of the non-BPD (#30, #50, #52, #56) and the BPD (#11, #14, #17, #18, #44) samples is shown by the boxplot. * indicates *p* < 0.05.

Both *Wnt5a^CAG^* and *Ror^Gli;GFP^* lungs represent a phenotype of arrested alveologenesis similar to the histological characteristic of human BPD. Expression or disruption of *WNT5a* in BPD lungs has not been previously reported. Therefore, to ascertain the relevance of our findings in the mouse to human BPD, we determined the expression of *WNT5a* in lungs of human patients who died with BPD. As shown in Figure 8, *WNT5a* was significantly lower in the BPD lungs (# 11, #14, #17, #18, #44) as compared to lungs from the non-BPD patients (#30, #50, #52, #56) who died due to non-pulmonary causes. No significant difference was observed in *ROR1* and *ROR2* expression.

## 4. Discussions

Late lung development that encompasses the periods of saccular and alveolar development are especially important as they include the cellular and molecular processes required for lung functional maturation as an efficient gas exchange organ at birth and throughout life. The role of WNT5a, a non-canonical WNT, in late lung development is unknown. In the present study, we generated and utilized conditional loss-of-function genetic models to examine the temporal-specific role of WNT5a in the saccular and alveolar phases of lung development. We demonstrate that finely tuned regulation of WNT5a is critical for both structural and cellular differentiation in the lung. In the saccular phase, WNT5a regulates differentiation of both mesodermal and epithelial lineages. Most importantly, we found that in the alveologenesis phase, inactivation of *Wnt5a* interrupts alveolar formation by disrupting differentiation and migration of the key mesodermally derived SCMF. The significance and relevance of these findings are highlighted by the fact that alveolar defects are a hallmark of many human neonatal and adult lung diseases.

**Figure 9:**
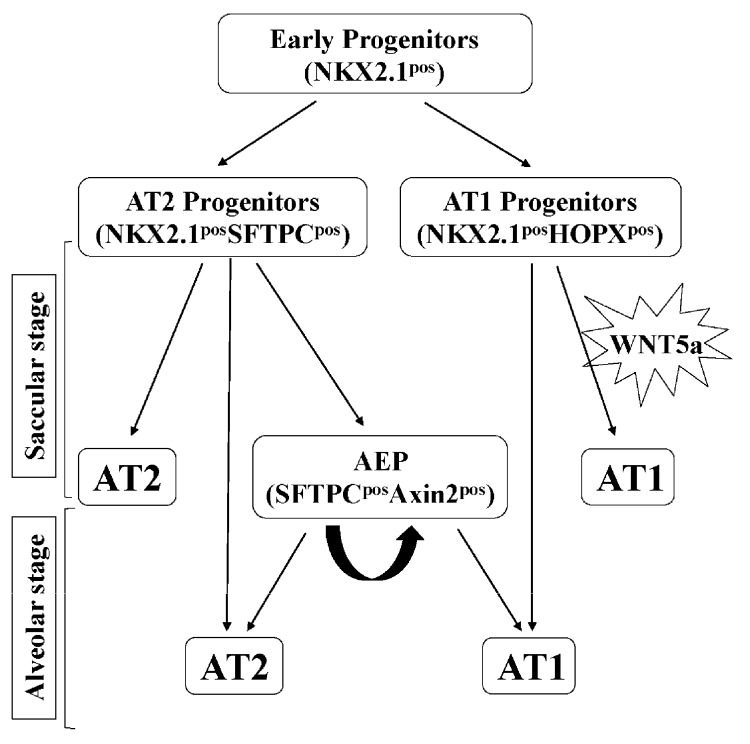
A simplified model indicating the role of WNT5a in AT1 and AT2 cell differentiation. During the saccular stage, WNT5a activity is required for differentiation of AT1 cells from NKX2.1^pos^HOPX^pos^ AT1 progenitors. During alveologenesis, WNT5a activity does not appear to be required for AT1 and AT2 cell differentiation. AEP: alveolar epithelial progenitors.

The key findings of the present study are that WNT5a regulation of late lung development is stage and cell-type specific. Conditional inactivation of *Wnt5a* in the saccular phase resulted in defects in structural development of the lung that appeared to be caused by failure in distal airway expansion (Figure 1). This is reminiscent of our previously reported results using a conventional germline *Wnt5a* loss-of-function model [10]. In the saccular model, we also found significantly reduced AT1 cell differentiation. Five of the seven tested AT1 cell markers were significantly decreased in the *Wnt5a^CAG^*lungs (Figure 2), reflecting an important role of WNT5a in AT1 cell differentiation during the saccular phase of lung development. Interestingly, expression of most AT1cell markers was not significantly affected by *Wnt5a* inactivation during alveologenesis (Figure 4), suggesting that the role of WNT5a in AT1 cell differentiation may be stage dependent. During the saccular phase of lung development, AT1 progenitors (NKX2.1^pos^HOPX^pos^) and AT2 progenitors (NKX2.1^pos^SFTPC^pos^) begin to give rise to mature AT1 and AT2 cells, respectively [2]. As lung development enters the alveolar phase, a subpopulation of AT2 cells maintains progenitor properties. These alveolar epithelial progenitors (AEP) are SFTPC^pos^AXIN2^pos^ and are capable of self-renewal or differentiation into mature AT1 and AT2 cells [32]. Results from the current study demonstrate that it is the saccular phase events, which are exquisitely sensitive to lack of WNT5a signaling (Figure 9). Interestingly, in adult lungs *Wnt5a* is expressed in the AT2 stem-cell niche (Axin2^pos^ AT2 cells), indicating a potential role for WNT5a in AT2 stem-cell maintenance [9]. Deletion of *Wnt5a* with *Tbx4^LME^-cre* was shown to moderately reduce AT2 stem cells but the reduction was not significant [9]. Recently Wu and colleagues showed that WNT5a represses the growth of alveolar epithelial progenitors in both lung-slice and organoid cultures [33]. Collectively, these data demonstrate a stage-dependent functional importance of WNT5a in lung development and stem-cell maintenance. 

The present study revealed that differentiation and morphogenesis of mesodermally derived cells are also sensitive to lack of WNT5a signaling in a temporally specific manner. *Wnt5a* inactivation reduced endothelial markers only in the saccular stage, but not during alveologenesis. Formation of capillary networks, which commences during the saccular phase, is regulated by the interaction between endothelial cells and pericytes. A recent study demonstrated that WNT5a from endothelial cells is important for pericyte recruitment [11]. Interestingly, our present study showed that expression of the pericyte marker *Pdgfrb* was reduced by *Wnt5a* inactivation during both the saccular and alveolar phases of lung development. Abnormal capillary distribution was also found in the germline deletion model of *Wnt5a* [10]. A number of previous studies have demonstrated that WNT5a induces endothelial cell migration through non-canonical WNT signaling pathways [15,34,35].

The major impact of *Wnt5a* inactivation during alveologenesis is on myofibroblast differentiation. As shown in Figure 5, multiple myofibroblast markers that included *Acta2, Tagln, Des, Dbn1, Tgfbi* and *Fbln1* were reduced in neonatal *Wnt5a^CAG^*lungs. In contrast, markers for other fibroblast cell types, such as lipofibroblasts (*Adrp*), was not significantly altered. This indicates that myofibroblasts are a major target of WNT5a signaling during alveologenesis, and *Wnt5a* deficiency disrupts their differentiation and migration. A key myofibroblast cell type with a critical role in alveologenesis is SCMF. In support of our findings, we found that blocking WNT5a signaling via targeting its receptors, *Ror* and *Ror2*, in SCMF (Figure 7) arrested alveologenesis. While the expression of myofibroblast markers was only moderately reduced in the *Ror^Gli;GFP^* lungs (Figure 7), this is likely because *Gli1cre^ERT2^* does not target all ACTA2^pos^ myofibroblasts (Figure 7, Panels D-F). Targeting *Rors* in SCMF also did not completely inhibit their differentiation, as some of the GFP^pos^ cells were still positive for ACTA2. Nevertheless, because of the critical role of SCMF, this level of gene-targeting was sufficient to cause a significant defect in alveologenesis, indicating the importance of WNT5a-ROR signaling in SCMF function during neonatal lung development.

Our recent study demonstrated that PDGFRa-mediated elastogenesis is important for lung maturation [25]. However, we found no evidence by RNA-seq analysis for alterations in *Wnt5a* expression in *Pdgfra* mutant SCMF. In addition, *Pdgfra* expression was not reduced in the *Wnt5a^CAG^* lungs (Figure 5). This indicates that PDGFRa signaling and WNT5a signaling may regulate alveologenesis via separate pathways. Interestingly, inactivation of *Wnt5a, Ror1 and Ror2,* as well as *Pdgfra* all decreased the elastogenic gene *Fbln1* (Figure 5 and Figure 7) [25], indicating that the two pathways may cross-talk over key mediators of alveologenesis, such as *Fbln1*. 

BPD is a morbid and sometimes lethal chronic lung disease in human prematurely born neonates [36]. Histological analysis of lung tissue from infants who died with BPD is consistent with a morphology characteristic of arrested alveolar formation [37]. While a number of molecules have been associated with BPD, causative factors in its pathogenesis remain unknown. A key reason is paucity of data on mechanisms of alveolar formation. The findings of the present study show that WNT5a is causally related to alveologenesis, the process that is arrested in human BPD. To examine the clinical relevance of the mouse findings, we show that *WNT5a* is also reduced in human BPD lung samples. While expression or the potential role of WNT5a in BPD has not been directly elucidated, multiple studies reported elevated nuclear β-catenin in both rodent hyperoxia BPD-phenocopy models and human BPD tissue [38,39]. Moreover, it has been shown that neonatal hyperoxia increases nuclear β-catenin and decreases AT2 to AT1 cell differentiation [40]. WNT5a inhibits β-catenin-mediated WNT signaling [6,12,28,33]. Thus, it is likely that reduced levels of WNT5a may contribute to increased canonical WNT signaling and pathogenesis of BPD. 

## Supplementary Materials

The following are available online at https://www.mdpi.com/2073-4409/9/2/384/s1, Figure S1: Craniofacial defects of E18 *Wnt5a^CAG^* embryos. Table S1: Expression of cell type specific markers during saccular and alveolar phases of lung development (References: LungGENS: https://research.cchmc.org/pbge/lunggens; [32,41,42]). Table S2: Changes of cell migration genes in *Pdgfra* deficient SCMF identified by RNAseq analysis (GSE126457, [25]. Table S3: Antibody information. Table S4: Sequences of real-time RT-PCR primers.

## References

[1] Shi W., Bellusci S., Warburton D. (2007). Lung Development and Adult Lung Diseases. Chest.

[2] Frank D.B., Penkala I.J., Zepp J.A., Sivakumar A., Linares-Saldana R., Zacharias W.J., Stolz K.G., Pankin J., Lu M., Wang Q. (2019). Early lineage specification defines alveolar epithelial ontogeny in the murine lung. Proc. Natl. Acad. Sci..

[3] Branchfield K., Li R., Lungova V., Verheyden J.M., McCulley D., Sun X. (2016). A three-dimensional study of alveologenesis in mouse lung. Dev. Biol..

[4] Boström H., Willetts K., Pekny M., Levéen P., Lindahl P., Hedstrand H., Pekna M., Hellström M., Gebre-Medhin S., Schalling M. (1996). PDGF-A Signaling Is a Critical Event in Lung Alveolar Myofibroblast Development and Alveogenesis. Cell.

[5] Lindahl P., Karlsson L., Hellström M., Gebre-Medhin S., Willetts K., Heath J.K., Betsholtz C. (1997). Alveogenesis failure in PDGF-A-deficient mice is coupled to lack of distal spreading of alveolar smooth muscle cell progenitors during lung development. Development.

[6] Li C., Li M., Li S., Xing Y., Yang C.-Y., Li A., Borok Z., De Langhe S., Minoo P. (2015). Progenitors of secondary crest myofibroblasts are developmentally committed in early lung mesoderm. Stem Cells.

[7] Kikuchi A., Yamamoto H., Kishida S. (2007). Multiplicity of the interactions of Wnt proteins and their receptors. Cell. Signal.

[8] Endo M., Nishita M., Fujii M., Minami Y. (2015). Insight into the Role of Wnt5a-Induced Signaling in Normal and Cancer Cells. Int. Rev. Cell Mol. Biol..

[9] Nabhan A.N., Brownfield D.G., Harbury P.B., Krasnow M.A., Desai T.J. (2018). Single-cell Wnt signaling niches maintain stemness of alveolar type 2 cells. Science.

[10] Li C., Xiao J., Hormi K., Borok Z., Minoo P. (2002). Wnt5a participates in distal lung morphogenesis. Dev. Boil..

[11] Yuan K., Shamskhou E.A., Orcholski M.E., Nathan A., Reddy S., Honda H., Mani V., Zeng Y., Ozen M.O., Wang L. (2019). Loss of Endothelium-Derived Wnt5a Is Associated With Reduced Pericyte Recruitment and Small Vessel Loss in Pulmonary Arterial Hypertension. Circulation.

[12] Baarsma H.A., Skronska-Wasek W., Mutze K., Ciolek F., Wagner D.E., John-Schuster G., Heinzelmann K., Günther A., Bracke K.R., Dagouassat M. (2017). Correction: Noncanonical WNT-5A signaling impairs endogenous lung repair in COPD. J. Exp. Med..

[13] Vuga L.J., Ben-Yehudah A., Kovkarova-Naumovski E., Oriss T., Gibson K.F., Feghali-Bostwick C., Kaminski N. (2009). WNT5A Is a Regulator of Fibroblast Proliferation and Resistance to Apoptosis. Am. J. Respir. Cell Mol. Boil..

[14] Kumawat K., Menzen M.H., Slegtenhorst R.M., Halayko A.J., Schmidt M., Gosens R. (2014). TGF-β-Activated Kinase 1 (TAK1) Signaling Regulates TGF-β-Induced WNT-5A Expression in Airway Smooth Muscle Cells via Sp1 and β-Catenin. Plos One.

[15] Loscertales M., Mikels A.J., Hu J.K.-H., Donahoe P.K., Roberts D.J. (2008). Chick pulmonary Wnt5a directs airway and vascular tubulogenesis. Development.

[16] Li C., Hu L., Xiao J., Chen H., Li J.T., Bellusci S., Delanghe S., Minoo P. (2005). Wnt5a regulates Shh and Fgf10 signaling during lung development. Dev. Boil..

[17] Nomi M., Oishi I., Kani S., Suzuki H., Matsuda T., Yoda A., Kitamura M., Itoh K., Takeuchi S., Takeda K. (2001). Loss of mRor1 Enhances the Heart and Skeletal Abnormalities in mRor2-Deficient Mice: Redundant and Pleiotropic Functions of mRor1 and mRor2 Receptor Tyrosine Kinases. Mol. Cell. Boil..

[18] Ho H.-Y.H., Susman M.W., Bikoff J.B., Ryu Y.K., Jonas A.M., Hu L., Kuruvilla R., Greenberg M.E. (2012). Wnt5a–Ror–Dishevelled signaling constitutes a core developmental pathway that controls tissue morphogenesis. Proc. Natl. Acad. Sci. USA.

[19] Mikels A.J., Nusse R. (2006). Purified Wnt5a protein activates or inhibits beta-catenin-TCF signaling depending on receptor context. PLoS Boil..

[20] Fukuda T., Chen L., Endo T., Tang L., Lu D., Castro J.E., Widhopf G.F., Rassenti L.Z., Cantwell M.J., Prussak C.E. (2008). Antisera induced by infusions of autologous Ad-CD154-leukemia B cells identify ROR1 as an oncofetal antigen and receptor for Wnt5a. Proc. Natl. Acad. Sci. U.S.A..

[21] Li C., Bellusci S., Borok Z., Minoo P. (2015). Non-canonical WNT signalling in the lung. J. Biochem..

[22] Ryu Y.K., Collins S.E., Ho H.-Y.H., Zhao H., Kuruvilla R. (2013). An autocrine Wnt5a-Ror signaling loop mediates sympathetic target innervation. Dev. Boil..

[23] Ahn S., Joyner A.L. (2004). Dynamic Changes in the Response of Cells to Positive Hedgehog Signaling during Mouse Limb Patterning. Cell.

[24] Bhattacharya S., Go D., Krenitsky D.L., Huyck H.L., Solleti S.K., Lunger V.A., Metlay L., Srisuma S., Wert S.E., Mariani T.J. (2012). Genome-Wide Transcriptional Profiling Reveals Connective Tissue Mast Cell Accumulation in Bronchopulmonary Dysplasia. Am. J. Respir. Crit. Care Med..

[25] Li C., Lee M.K., Gao F., Webster S., Di H., Duan J., Yang C.-Y., Bhopal N., Peinado N., Pryhuber G. (2019). Secondary crest myofibroblast PDGFRα controls the elastogenesis pathway via a secondary tier of signaling networks during alveologenesis. Development.

[26] McGowan S.E., Grossmann R.E., Kimani P.W., Holmes A.J. (2008). Platelet-Derived Growth Factor Receptor-Alpha-Expressing Cells Localize to the Alveolar Entry Ring and Have Characteristics of Myofibroblasts During Pulmonary Alveolar Septal Formation. Anat. Rec. Adv. Integr. Anat. Evol. Boil..

[27] Yamaguchi T.P., Bradley A., McMahon A.P., Jones S. (1999). A Wnt5a pathway underlies outgrowth of multiple structures in the vertebrate embryo. Development.

[28] Rieger M.E., Zhou B., Solomon N., Sunohara M., Li C., Nguyen C., Liu Y., Pan J.-H., Minoo P., Crandall E.D. (2016). p300/β-Catenin Interactions Regulate Adult Progenitor Cell Differentiation Downstream of WNT5a/Protein Kinase C (PKC)*. J. Boil. Chem..

[29] Thurlbeck W.M. (1967). Measurement of pulmonary emphysema. Am. Rev. Respir. Dis..

[30] D’Hulst A.I., Vermaelen K.Y., Brusselle G.G., Joos G.F., Pauwels R.A. (2005). Time course of cigarette smoke-induced pulmonary inflammation in mice. Eur. Respir. J..

[31] Moiseenko A., Kheirollahi V., Chao C.-M., Ahmadvand N., Quantius J., Wilhelm J., Herold S., Ahlbrecht K., Morty R.E., Rizvanov A.A. (2017). Origin and characterization of alpha smooth muscle actin-positive cells during murine lung development. Stem Cells.

[32] Frank D.B., Peng T., Zepp J.A., Snitow M., Vincent T.L., Penkala I.J., Cui Z., Herriges M.J., Morley M.P., Zhou S. (2016). Emergence of a Wave of Wnt Signaling that Regulates Lung Alveologenesis by Controlling Epithelial Self-Renewal and Differentiation. Cell Rep..

[33] Wu X., Dijk V., Ng-Blichfeldt J.P., Bos I.S.T., Ciminieri C., Königshoff M., Kistemaker L.E., Gosens R., Wu X., Van Dijk E.M. (2019). Mesenchymal WNT-5A/5B Signaling Represses Lung Alveolar Epithelial Progenitors. Cells.

[34] Cornett B., Snowball J., Varisco B.M., Lang R., Whitsett J., Sinner D. (2013). Wntless is required for peripheral lung differentiation and pulmonary vascular development. Dev. Boil..

[35] Cheng C.-W., Yeh J.-C., Fan T.-P., Smith S.K., Charnock-Jones D.S. (2008). Wnt5a-mediated non-canonical Wnt signalling regulates human endothelial cell proliferation and migration. Biochem. Biophys. Res. Commun..

[36] Davidson L.M., Berkelhamer S.K. (2017). Bronchopulmonary Dysplasia: Chronic Lung Disease of Infancy and Long-Term Pulmonary Outcomes. J. Clin. Med..

[37] Husain A.N., Siddiqui N.H., Stocker J. (1998). Pathology of arrested acinar development in postsurfactant bronchopulmonary dysplasia. Hum. Pathol..

[38] Sucre J.M., Deutsch G.H., Jetter C.S., Ambalavanan N., Benjamin J.T., Gleaves L.A., Millis B.A., Young L.R., Blackwell T.S., Kropski J.A. (2018). A Shared Pattern of β-Catenin Activation in Bronchopulmonary Dysplasia and Idiopathic Pulmonary Fibrosis. Am. J. Pathol..

[39] Dasgupta C., Sakurai R., Wang Y., Guo P., Ambalavanan N., Torday J.S., Rehan V.K. (2009). Hyperoxia-induced neonatal rat lung injury involves activation of TGF-{beta} and Wnt signaling and is protected by rosiglitazone. Am. J. Physiol. Cell. Mol. Physiol..

[40] Xu W., Xu B., Zhao Y., Yang N., Liu C., Wen G., Zhang B. (2015). Wnt5a reverses the inhibitory effect of hyperoxia on transdifferentiation of alveolar epithelial type II cells to type I cells. J. Physiol. Biochem..

[41] Taylor S.K., Sakurai R., Sakurai T., Rehan V.K. (2016). Inhaled Vitamin D: A Novel Strategy to Enhance Neonatal Lung Maturation. Lung.

[42] Little D.R., Gerner-Mauro K.N., Flodby P., Crandall E.D., Borok Z., Akiyama H., Kimura S., Ostrin E.J., Chen J. (2019). Transcriptional control of lung alveolar type 1 cell development and maintenance by NK homeobox 2-1. Proc. Natl. Acad. Sci. U.S.A..

