# Supplementary material for: WNT5a-ROR Signaling Is Essential for Alveologenesis
Source: Cells. 2020 Feb 7;9(2):384. doi: 10.3390/cells9020384 (PMC7072327; doi:10.3390/cells9020384)
Supplement: Supplementary file 1 [file cells-09-00384-s001.pdf]

WNT5a-ROR Signaling Is Essential for Alveologenesis

Supplementary Materials:

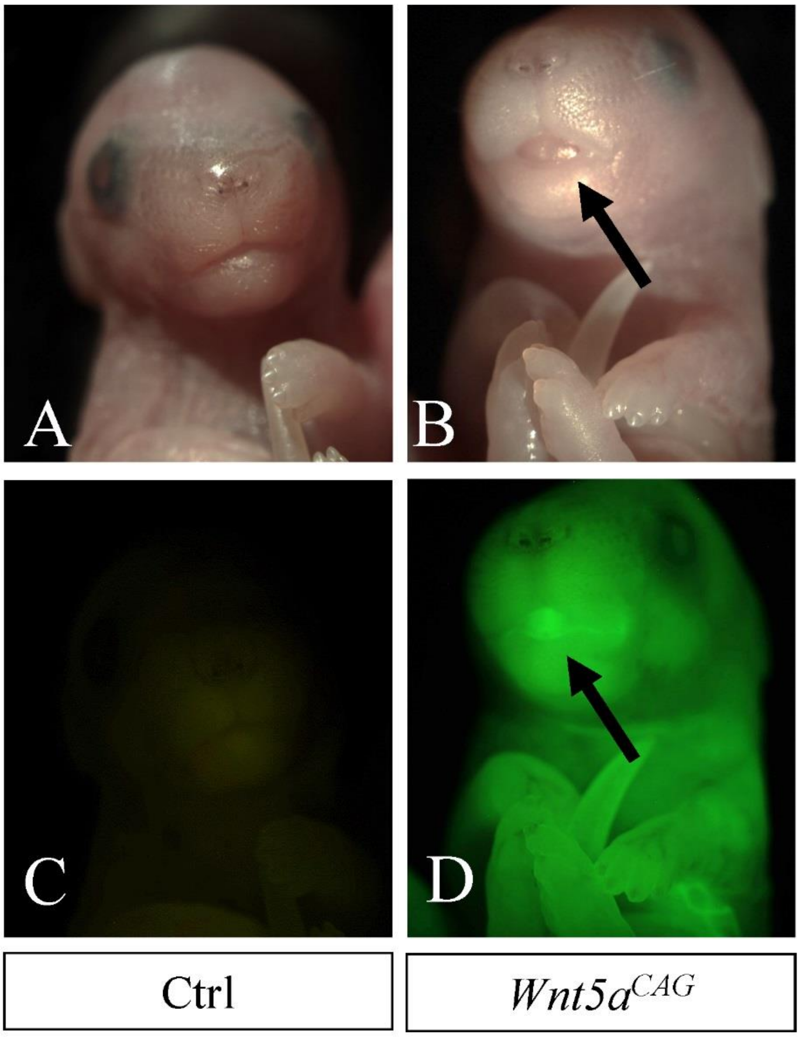

Figure S1: Craniofacial defects of E18 *Wnt5a*^{CAG} embryos.

A-D: Frontal view of E18 control (A, C) and *Wnt5a*^{CAG} (B, D) craniofacial phenotype. Panels A & B show the bright field images and Panels C & D show the green fluorescent images of control and *Wnt5a*^{CAG} embryos, respectively. Arrow indicates the "open" mouth phenotype in *Wnt5a*^{CAG}.

Table S1: Expression of cell type specific markers during saccular and alveolar phases of lung development.
Saccular phase cell specificity is based on mouse E18.5 scRNA-seq in LungGENS (LungGENS: https://research.cchmc.org/pbge/lunggens). Alveolar phase cell specificity is based on mouse P7 scRNA-seq in LungGENS for endothelial and myofibroblast markers, and publications [32,41,42] listed in the table for AT1 and AT2 markers.

Gene Symbol	Gene Name	Saccular Phase	Alveolar Phase (reference)
Aqp5	Aquaporin 5	AT1	AT1 (32)
Cav1	Caveolin 1	AT1, EC	AT1 (42)
Emp2	Epithelial Membrane Protein 2	AT1	AT1 (42)
Hopx	HOP Homeobox	AT1	AT1 (32)
Rage (Ager)	Advanced Glycosylation End-Product Specific Receptor	AT1	AT1 (42)
Rtkn2	Rhotekin 2	AT1, Ciliated Cells	AT1 (42)
Pdpn	Podoplanin	AT1	AT1 (32)
Sftpc	Surfactant Protein C	AT2	AT2 (32)
Sftpb	Surfactant Protein B	AT2	AT2 (41)
Abca3	ATP Binding Cassette Subfamily A Member 3	AT2, Club Cells	AT2 (32)
Cd31(Pecam1)	Platelet And Endothelial Cell Adhesion Molecule 1	EC	EC
Emcn	Endomucin	EC	EC
Flk1 (KDR)	Kinase Insert Domain Receptor	EC, Club Cells	none
Tek	TEK Receptor Tyrosine Kinase	EC	EC
Acta2	Actin Alpha 2, Smooth Muscle	MF/SMC	MF/SMC
Tagln	Transgelin	MF/SMC	MF/SMC
Des	Desmin	none	MF/SMC
Dbn1	Drebrin 1	MF/SMC	MF/SMC
Tgfbi	Transforming Growth Factor Beta Induced	MF/SMC	MF/SMC
Fbln1	Fibulin 1	MF/SMC, Ciliated Cells	MF/SMC
Eln	Elastin	MF/SMC, LipoFB/MatrixFB	MF/SMC
Col1a1	Collagen Type I Alpha 1 Chain	LipoFB/MatrixFB	MatrixFB
Pdgfra	Platelet Derived Growth Factor Receptor Alpha	MF/SMC	MF/SMC
Pdgfrb	Platelet Derived Growth Factor Receptor Beta	MF/SMC	none

Abbreviations: AT1: Alveolar epithelial type 1, **AT2:** Alveolar epithelial type 2, **EC:** Endothelial Cells, **MF:** Myofibroblasts, **SMC:** Smooth Muscle Cells, **LipoFB**: Lipofibroblasts, **MatrixFB**: Matrix fibroblasts, **none**: -log(p-value)<2.996 for most differentially expressed cell type.

Table S2: Changes of cell migration genes in *Pdgfra* deficient SCMF identified by RNA-seq analysis (GSE126457, [25]).

Gene	Fold Changes	P-value	FDR
Actc1	-7.9	0.00E+00	0.00E+00
Bmp2	2.94	4.25E-39	2.38E-37
Meox2	-7.06	6.50E-24	2.37E-22
Lum1	-14.74	1.55E-40	9.06E-39
Cyr61	2.27	0.00E+00	0.00E+00
Cnn1	-9.68	3.57E-168	7.53E-166
Fgf18	-2.22	6.32E-04	3.96E-03

Table S3: Antibody information (*: discontinued).

Anitbody & Reagents	Company	Catelog #
Mouse anti-GFP	Santa Cruz, CA	SC-9996
Rabbit anti-ACTA2	Novus, CO	NB600531*
Rabbit anti-ACTA2	Millipore, MA	ABT1487
Goat anti-TAGLN	Abcam, MA	AB10135
Rabbit anti-HOPX	Santa Cruz, CA	SC-30216*
Rabbit anti-SFTPC	Millipore, MA	AB3786
Hamster anti-PDPN	DSHB	8.1.1

Table S4: Sequences of real-time RT-PCR primers.

**Gene name (mouse)**	**realtime RT-PCR Primer Sequences**
Emcn	5'-TGC TTC AAG CGA CTG TTC TTT-3'<br>5'-GCC GGT GTT GGA ATA GAG TC-3'
Flk1	5'-CAGTGGTACTGGCAGCTAGAA-3'<br>5'-ACAAGCATACGGGCTTGTTT-3'
Cd31	5'-GAGTACGAGGTGAAGGTGCAT-3'<br>5'-CGCCTTCTGTCACCTCCTT-3'
Tek	5'-CAT AGG AGG AAA CCT GTT CAC-3'<br>5'-GCC CCC ACT TCT GAG CTT-3'
Acta2 (aSMA)	5'- CCCACCCAGAGTGGAGAA-3'<br>5'- ACATAGCTGGAGCAGCGTCT-3'
Tagln	5'-CCTTCCAGTCCACAAACGAC-3'<br>5'-GTAGGATGGACCCTTGTTGG-3'
Desmin (Des)	5'-GCG TGA CAA CCT GAT AGA CG-3'<br>5'-TGG AT TCC TCC TGT AGT TTG G-3'
Dbn1	5'-CCT GCA CCC TTC AAC CAC-3'<br>5'-GAA TAG GAG TGG GTG CCA TC-3'
Tgfbi	5'-GCT GCG AGT CTT TGT TTA TCG-3'<br>5'-CCT CTT ATC ATG GGC AGC A-3'
Eln	5'-GCTGCTGCTAAGGCTGCTAA-3'<br>5'-AGCACCTGGGAGCCTAACTC-3'
Col1a1	5'-CAT GTT CAG CTT TGT GGA CCT-3'<br>5'-GCA GCT GAC TTC AGG GAT GT-3'
Fbln1	5'-GTGCAAGGCTGGCTTCTATT-3'<br>5'-GATAGCGCTGGCACTCGT-3'
Pdgfra	5'-GTCGTTGACCTGCAGTGGA-3'<br>5'-CCAGCATGGTGATACCTTTGT-3'
Pdgfrb	5'-TCAAGCTGCAGGTCAATGTC-3'<br>5'-CCATTGGCAGGGTGACTC-3'
Ng2	5'-CTTGGCCTTGTTGGTCAGAT-3'<br>5'-CACCTCCAGGTGGTTCTCC-3'
Adrp	5'-CCTCAGCTCTCCTGTTAGGC-3'<br>5'-CACTACTGCTGCTGCCATTT-3'

**Gene name (human)**	**Realtime RT-PCR Primer Sequences**
WNT5a	5'-CGTCTGGAAGCAGACGTTTC-3'<br>5'-TCACGCCTCCTGATCTCC-3'
ROR1	5'-GCAAGGGGAAATAGAAAATCAG-3'<br>5'-GAAGGAATGGCGAACTGAGA-3'
ROR2	5'-CCCCTCATTAACCAGCACAA-3'<br>5'-TTCCCAAACCGGTCCTCT-3'

**Gene name (mouse)**	**realtime RT-PCR Primer Sequences**
Sftpb (SpB)	5'-AACCCCACACCTCTGAGAAC-3'<br>5'-GTGCAGGCTGAGGCTTGT-3'
Sftpc (Spc)	5'-GGTCCTGATGGAGAGTCCAC-3'<br>5'-GATGAGAAGGCGTTTGAGGT-3'
Abca3	5'-TGT GGC GAC TGA GAA CCT T-3'<br>5'-CAC TGA TGT CCG GTT CTG C-3'
Aqp5	5'-CAGACCTCAGAGATTGTGAAG-3'<br>5'- CAGAAATAAATAAGATGGCAC-3'
Caveolin1 (Cav1)	5'-CCA GGG AAA CCT CCT CAG A-3'<br>5'-CCG GAT GGG AAC AGT GTA GA-3'
Emp2	5'-AAG CAG GGA GAG AGG TTC GT-3'<br>5'-GCT CCG ATC ATG ACA CAC AG-3'
Hopx	5'-ACCACGCTGTGCCTCATC-3'<br>5'-GCGCTGCTTAAACCATTTCT-3'
Rage	5'-GTGTCGGGCAACTAACAGG-3'<br>5'-CTGGCTTCCCAGGAATCTG-3'
Rtkn2	5'-TGTATGGCAACGTGTGCTG-3'<br>5'-CCAGACCTTTTCCTGTTTGC-3'
Pdpn(T1a)	5'-CAGTGTTGTTCTGGGTTTTGG-3'<br>5'-TGGGGTCACAATATCATCTTCA-3'
Wnt5a	5'-TAA GCC CGG GAG TGG CTT TGG-3'<br>5'-GGG CGA AGG AGA AAA ACG TGG-3'
Actc1	5'-CCTCTCTGGAGAAGAGCTATGA-3'<br>5'-AATGAAAGAGGGCTGGAAGAG-3'
Bmp2	5'-AGATCTGTACCGCAGGCACT-3'<br>5'-GTTCCTCCACGGCTTCTTC-3'
Meox2	5'-AAT CTA GAC CTC ACT GAA AGA CAG G-3'<br>5'-CTT GCT GTC CCC CTT TGA-3'
Lum	5'-CAG CAA CAT TCC GGA TGA G-3'<br>5'-TCA TTG TGA GAT AAA CGC AGG T-3'
Cyr61	5'-GGATCTGTGAAGTGCGTCCT-3'<br>5'-CTGCATTTCTTGCCCTTTTT-3'
Cnn1	5'-CGGCTTGTCTGCTGAAGTAA-3'<br>5'-ACCCCCTCAATCCACTCTCT-3'
Fgf18	5'-AGGACGGGGACAAGTATGC-3'<br>5'-GGACTTGACTCCCGAAGGT-3'
Tbp	5'-CAGCCAAGATTCACGGTAGAT-3'<br>5'-CCAATGACTCCAATGACCCCT-3'
